# From Child Abuse to Developing Borderline Personality Disorder Into Adulthood: Exploring the Neuromorphological and Epigenetic Pathway

**DOI:** 10.7759/cureus.9474

**Published:** 2020-07-30

**Authors:** Pranita Mainali, Tehrima Rai, Ian H Rutkofsky

**Affiliations:** 1 Psychiatry, Washington DC Veterans Affairs Medical Center, Washington, DC, USA; 2 Psychiatry, California Institute of Behavioral Neurosciences & Psychology, Fairfield, USA; 3 Pediatrics, California Institute of Behavioral Neurosciences & Psychology, Fairfield, USA

**Keywords:** child abuse, early life stressor, borderline personality disorder, adverse childhood experience

## Abstract

Borderline personality disorder (BPD) is one of the most common personality disorders seen in the general population. Among multiple identified risk factors, one of the most influential elements is exposure to an adverse childhood experience in terms of emotional, physical, or sexual abuse. A cascade of neuromorphological and epigenetic changes occurs in response to these childhood stressors, which may have a strong link to the development of BPD. PubMed and Google Scholar were searched for articles relevant to child abuse and the development of BPD. The search was not restricted to any time frame or geographic location. Significant epigenetic and neuromorphological changes are seen with child abuse, contributing to the development of BPD. Chronic stressors lead to hypothalamic-pituitary axis (HPA) activation, releasing cortisol that acts on the prefrontal cortex, amygdala, and hippocampus, producing the behavioral patterns seen in BPD. Overstimulation of gray matter leads to permanent neuromorphological changes, which can be visualized in functional MRI/brain scans. Hypermethylation of messenger ribonucleic acid in various sites suggests the impact of child abuse on the genetic level. Interestingly, the prevalence of BPD is seen equally in both genders but is diagnosed more frequently in females because they tend to be more likely to seek help. Understanding the impact of early age life stressors into adulthood calls for serious focus on early diagnosis and intervention. This implies the need for more studies in patients with BPD with or without any childhood traumatic experience and a better understanding of the changes that occur biopsychologically and genetically in response to trauma.

## Introduction and background

Nearly 7.5 million children are victims of abuse in the United States (US) annually, where 74.9% are victims of neglect, 18.3% are physically abused, 8.6% are sexually abused, and 7.1% are psychologically maltreated [1]. Children younger than one year, especially males, have the highest rate of child abuse. Nearly 50% of childhood fatalities occur in the first year of life [2]. However, abuse remains a concern in all age groups, with 27% of victims being younger than three years of age, and 20% are aged three to four years [3]. Child abuse is a public concern, as it is estimated that five to six children die every day because of domestic violence, physical abuse, sexual trauma, or neglect [4]. About 80% of victims develop a psychiatric illness, behavioral, and emotional issues before the age of 21 years [5]. Recent literature has suggested a significant relationship between child abuse and its persistence with the development and severity of maladaptive personality traits into adulthood [6,7]. Among all personality disorders, 30% to 90% of patients who meet the criteria for borderline personality disorder (BPD) have a history of child abuse or trauma [8].

BPD is a common personality disorder seen in 1% to 3% of the general population, 10% in outpatient settings, 20% in inpatient settings, and 9% to 27% in the emergency department [9,10]. It is characterized by a marked pervasive pattern of emotional dysregulation, impulsive behavior, identity disturbances, and interpersonal conflicts [11]. Predominantly, it is seen three to four times higher in females in various clinical settings, even though the gender prevalence remains nearly equal in the community [12]. A study has shown that early biological and neurological stressors lead to DNA methylation, which impairs usual brain functioning, interfering with emotional regulation and stability, impulse control, coping skills, interpersonal skills, cognition, and other core skills, as seen in BPD [13]. However, limited studies have been conducted to establish a clear correlation between child abuse or trauma and the development of BPD later in life. The fact that men are less likely to seek help also creates a challenge to understanding the prevalence and severity of BPD with child abuse [14]. The purpose of this study is to explore the possible epigenetic modifications and neurobiological changes due to early life stressors and related to the development of BPD into adulthood. This study also seeks to assess these neuromorphological changes and understand the sex distribution of BPD.

## Review

Method

PubMed and Google Scholar were searched for articles relevant to child abuse and the development of BPD later in life. A comprehensive search was done to understand the association of epigenetic and neurobiological effects of childhood trauma with the development of BPD, along with the impact of duration and severity on both sexes. Medical Subject Heading (MeSH) terms used were “child abuse”, “adult survivor of child abuse”, and “child abuse, sexual” which returned 399 peer-reviewed articles; “early life stressor” returned 533 articles, “childhood trauma” returned 16,657 publications, and “borderline personality disorder” returned 6,439 publications. Keeping the search relevant to the topic, MeSH terms were combined with other phrase choices consisting of “childhood trauma and borderline personality disorder” which returned 204 articles; “trauma exposure in children and borderline personality disorder” returned two systematic reviews, “borderline personality disorder and gender” returned 525 articles, and combining all the above-mentioned MeSH terms yielded eight articles. All articles were reviewed including the abstracts for the restricted one. The search was not restricted to any timeframe or geographic location.

Result

This review shows that any form of child abuse can lead to long-term neurobiological and permanent morphological changes in the brain of the victim. Overactivation of the hypothalamic-pituitary axis (HPA) leads to excess cortisol production. This mechanism consistently prepares the body for a flight or fight response and misinterprets standard environmental signals as a threat. Overstimulation of gray matter leads to a reduction in the volume of the hippocampus, activation of the amygdala, and impairment of the prefrontal, frontal limbic, and parietal areas. All these changes lead to the personality changes seen with individuals with BPD. BPD may be recorded more frequently in women who tend to seek psychopharmacological treatment, but in reality, both sexes are equally affected [15]. Due to differences in the nature and presentation of the condition among men and women, males with BPD are more frequently incarcerated due to aggressive or drug-seeking behavior, and females are more often evaluated in healthcare settings [16]. It is important to evaluate every abused child regardless of sex, as the impact is not only seen clinically but also on the genetic level, triggering hypermethylation of messenger ribonucleic acid in various sites, as reflected in individuals with BPD [17,18].

Neurobiological variations

Child abuse leads to long-term repercussions as stressors trigger chronic “hyperarousal” of the HPA, which mediates cascades of neurohormonal changes in response [19]. The paraventricular nucleus in the hypothalamus releases corticotropin-releasing factor, which stimulates corticotrophin cells to release adrenocorticotropic hormone (ACTH) [20]. ACTH then acts on the adrenal glands to stimulate the secretion of cortisol, the primary stress hormone. Cortisol functions as the major hormone for the fight or flight response; it activates the autonomic nervous system (ANS), leading to an arousal state through the sympathetic nervous system [21]. Under chronic stress, unregulated cortisol secretion could lead to disruption of the ANS, as supported by a meta-analysis conducted by Koenig et al. [22]. The study draws attention to the disinhibition symptoms of BPD like impulsivity, self-harming behavior, emotional dysregulation, and its link to the lower resting-state vagal tone measured by vagally mediated heart rate variability.

The hyperarousal of the hippocampus by cortisol leads to misinterpretation of signals perceived from the natural environment, individuals, and situations, as constant threats and sends the wrong message to the amygdala. On the other hand, activation of the amygdala, which regulates fear and aggression, results in unstable and unpredictable intense emotional turmoil in response to minor stress with a more extended latency for returning to baseline; this is common in BPD. Overfunctioning of the prefrontal cortex explains the loss of rationality, reasoning, and decision-making capacity in BDP (Doctoral Dissertation: Human C. Neuropsychological Deficits in Borderline Personality Disorder, University of Johannesburg; 1998) [23,24].

**Figure 1:**
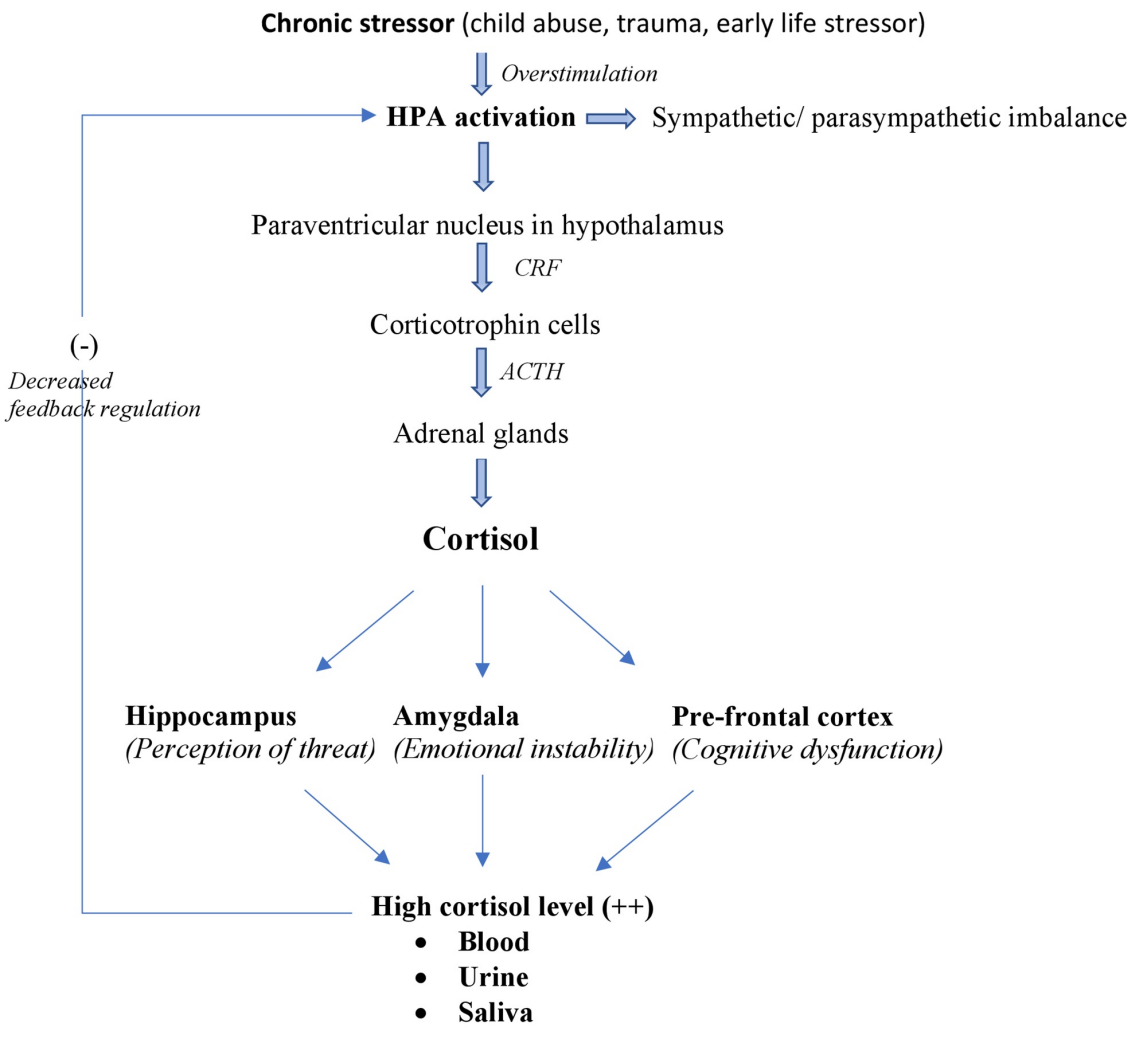
Impact of the chronic childhood stress on HPA activation HPA, hypothalamic-pituitary axis; CRF, corticotrophin-releasing factor; ACTH, adrenocorticotrophic hormone.

Many studies have shown an increase in urinary, salivary, or blood cortisol levels in individuals with BPD with or without comorbid posttraumatic stress disorder (PTSD), which is a hallmark of HPA activation. In 2002, Rinne et al. conducted a study in 39 patients with or without childhood trauma and with a BPD diagnosis [25]. The study showed a higher level of ACTH (and hence cortisol level) in the blood of patients with BPD, who had also sustained early life stressors. In 2007, Wingenfeld et al. conducted a study on 21 women with BPD and 24 controls without BPD with the conclusion that overnight urinary cortisol level was much higher in women with BPD in comparison to those in the control group [26]. Similarly, another study conducted by Lieb et al. linked BPD to overactivation of the adrenal glands and decreases in the HPA regulatory feedback mechanism, as evidenced by a significant elevation in salivary cortisol level in 23 unmedicated women with BPD in comparison to 24 control women (Figure 1) [27].

Neuromorphological variation

Many studies have shown visual evidence of morphological changes, mainly in the hippocampal area, amygdala, hypothalamus, and prefrontal cortex in patients with BPD [28-30]. In 2001, Herpertz et al. conducted a study to scan brain activity using functional MRI in six women with BPD and without other psychiatric comorbidities with six matched control women [31]. The study showed increased blood oxygenation levels on both sides of the amygdala along with activation of the medial and inferolateral prefrontal cortex. This association explains the emotional instability in BPD patients even with low stressors and increased latency for the emotion to return to baseline. Similarly, in 2013, an analysis by Kuhlmann et al. on 30 BPD patients and 33 matched controls showed decreased hippocampal volumes in those with BPD along with increased volume in the hypothalamus. Remarkably, the size of the hypothalamus has a direct relationship with exposure to childhood trauma in patients with BPD [32].

Soloff et al. in 2012 conducted a more extensive study, including 68 participants with BPD (16 men and 52 women) and 52 healthy control group (28 men and 24 women), with suicidal attempts or suicidal behaviors [33]. A structural MRI scan showed a significant decrease in gray matter in the left insula, right mid-superior temporal gyrus, right mid-inferior orbitofrontal gyrus, right insular cortex, left fusiform gyrus, left lingual gyrus, and right parahippocampal gyrus in patients with BPD and lethal suicidal attempts. Using three-dimensional (3D) MRI, Irle et al. found that 30 women with BPD and severe childhood trauma had a drastic reduction of hippocampal volume by 17% and a reduction in parietal cortex volume by 11% in comparison to control group [34]. This study showed a direct proportional correlation between the severity of trauma exposure in childhood and the amount of volume reduction in the mentioned areas of the brain. Therefore, in line with the findings of a study by Dannlowski et al., there is a possibility that the symptoms of BPD may be due to overstimulation of the hippocampal area leading to the gradual reduction in gray matter volume, as is commonly seen in patients who are victims of early traumatization and PTSD (Table 1) [31-35].

**Table 1:** Summary of neuromorphological changes seen in patients with borderline personality disorder. BPD, borderline personality disorder; CTQ, childhood trauma questionnaire; 3D, three dimensional.

Author	Study year	Implications	Sample size	Result
Dannlowski et al. [35]	2012	Overstimulation of gray matter leads to a decrease in hippocampal volume = loss of memory, flexible cognition, and social behavior	148 healthy subjects screened for childhood maltreatment using CTQ	Functional MRI analysis showed reduced gray matter volume in the hippocampus, insula, orbitofrontal cortex, anterior cingulate gyrus, and caudate in subject with high CTQ score
Herpertz et al. [31]	2001	Amygdala activation = intense emotion to low stressor; increased attention to emotionally activating external stimuli	Six BPD females without other psychiatric comorbidities and six matched control females	Functional MRI showed increased blood oxygenation level on both sides of the amygdala; activation of medical and inferolateral prefrontal cortex in BPD
Kuhlmann et al. [32]	2013	Decreased hippocampal volume = increased stress and glucocorticoid activation; increased hypothalamus volume = overstimulation of central stress regulation	30 patients with BPD (unmedicated), 33 healthy controls	Decrease in hippocampal volume with BPD; increase in the hypothalamus compared to control
Soloff et al. [33]	2012	Structural abnormality: in prefrontal and frontolimbic region = emotional instability, cognitive functioning impairment, and impulsive behavior	68 with BPD (16 males, 52 females) and 52 healthy controls (28 males, 24 females)	Diminished gray matter concentration in the left insula, right mid-superior temporal gyrus, right mid-inferior orbitofrontal gyrus, right insular cortex, left fusiform gyrus, left lingual gyrus, and right parahippocampal gyrus in BPD with lethal suicidal attempts
Irle et al. [34]	2005	Parietal atrophy = misinterpretation of sensory/visual information and constant perception of threat to normal stimuli; abnormality in temporoparietal cortex = psychotic symptoms	30 BPD females with a history of childhood trauma and 25 matched healthy controls	3D structural MRI showed decreased in hippocampal area by 17% and in the parietal region by 11% in females with BPD and trauma history

Epigenetic modifications with childhood maltreatment

Along with the overstimulation of the HPA axis, there is increased methylation of glucocorticoid receptor gene NR3C1, as observed by Martin-Blanco et al. in an analysis conducted in 2014 [36]. Among 281 subjects with BPD, higher NR3C1 methylation was seen in patients with BPD who had exposure to childhood trauma [32]. Similarly, Dammann et al. analyzed DNA methylation in a patient with BPD and compared the results with the control group [17]. Of 14 neurogenes, they found increased methylation of HTR2A (by 0.8%), NR3C1 (by 1.8%), MAOA (by 1.5%), MAOB (by 1.4%), and soluble COMT (S-COMT). Overall, the methylation of these genes was 1.7% higher in patients with BPD and childhood trauma (p < 0.0001) in comparison to patients in the control group. A similar finding was reported by Teschler et al., who described 1.26-fold higher methylation in an analyzed gene associated CpG sites in the blood sample of 24 female BPD patients in comparison to 11 female healthy controls [18].

**Figure 2:**
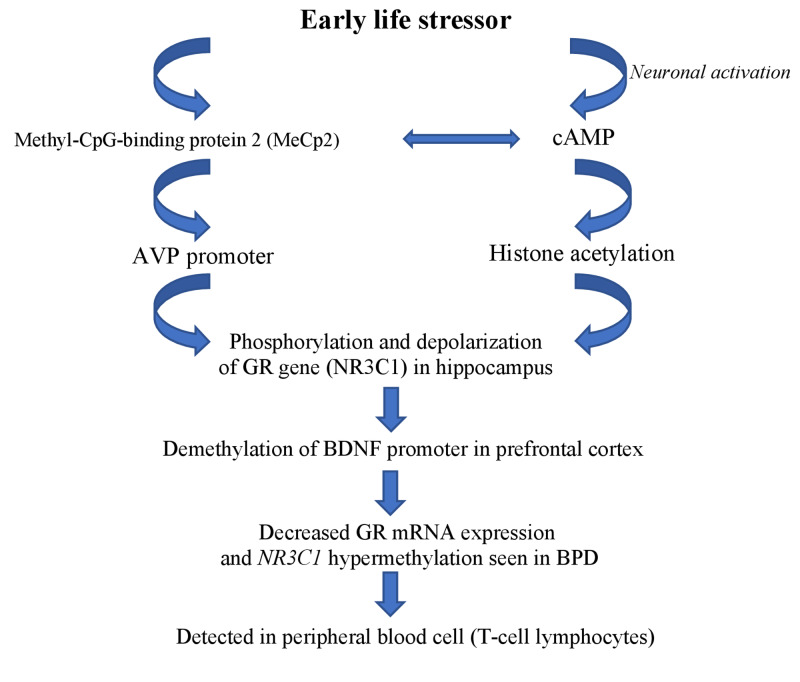
Environmental stressor leading to epigenetic modification. AVP, arginine vasopressin gene; cAMP, adenosine 3’,5’-cyclic monophosphatase; BDNF, brain-derived neurotrophic factor gene; GR gene, glucocorticoid receptor gene; mRNA, messenger ribonucleic acid; NR3C1, nuclear receptor subfamily 3, group C, member 1; BPD, borderline personality disorder.

The influence of environmental stressors leading to epigenetic modification was summarized by Gescher et al. in a meta-analysis published in 2018 (Figure 2) [37].

Gender prevalence

An analysis by Sansone and Sansone proposed that men with BPD are more likely to be found incarcerated due to violent temperament or substance use disorders, while women with BPD are more likely to be seen in healthcare settings for seeking treatment for eating disorders, mood disorders, anxiety, or PTSD [16]. Tadić et al. investigated the gender difference between axis I and axis II patients with BPD diagnosis. Among 110 women and 49 men with BDP, in axis I, men had more alcohol dependence (65% vs. 43%), and more women suffered from anorexia nervosa (21% vs. 4%). Men presented more often with “anger issues” (74% vs. 49%), and women presented more often with “emotional instability” (94% vs. 82%) [38].

Zanarini et al. discussed a strong correlation between gender and expression of axis II comorbidities. The analysis concluded that more males with BPD diagnosis meet the criteria for paranoia, passive-aggressive, sadistic, narcissistic, or antisocial personality disorders [39]. Interestingly, Marchetto, in 2006, found no significant gender difference with regards to self-harming behavior such as repetitive skin cutting [40]. Although there are differences in the presentation of BPD in men and women, the difference in the level of impairment remains insignificant [15]. However, as women with BPD tend to seek more pharmacotherapy and psychotherapy in their lifetime as compared to men with BPD, this might account for the higher prevalence of BPD seen in females (3:1) as opposed to males [41,42].

This literature review sheds light on the impact of child abuse and early life stressors on the neurogenetic level, raising concerns for cautiousness in screening children with any form of abuse. The consequences of early life stress are multidirectional; however, with a focus on developing BPD into adulthood, initial screening and diagnosis can lead to timely intervention and prevention in the long run. This paper encapsulates the neurogenetic and morphological changes seen in both sexes, as detected in serum studies, through genetic testing and brain scans. This can also be applied to guiding BPD diagnosis in the various developmental stages of childhood. Additionally, this knowledge can be used to understand the impact of therapy or trauma-focused intervention.

Understanding the link between child abuse and the development of any type of personality disorder can change the approach to such situations. Long-term follow-up from the day of screening and diagnosis is equally crucial, along with developing case-specific individual treatment modalities. These interventions can lead to preventing or mitigating some of the molecular-level changes that lead to the development of BPD. Child abuse in itself is a public concern, and understanding the short-term and long-term mental and physical consequences can be valuable in improving the quality of life of victims immediately and in the long run.

This study is not without limitations. This review focuses mainly on understanding the correlation between the early life stressor of child abuse and the development of BPD later into adulthood. This study does not specify the various types of trauma experiences (emotional, physical, or sexual abuse) and does not examine exposure to each type of abuse and their effect on the genetic and neurological levels. Moreover, it does not account for various demographic variables such as the age of the child when exposed to trauma, duration of exposure, stressor severity, family history, and socioeconomic history, factors that play a vital role in the development of personality disorders. 

## Conclusions

Child abuse is a risk factor for the development of BPD and is a public concern. Understanding the impact of early age negative life stressors on adulthood calls for serious focus on early diagnosis and intervention. This implies the need for more research focusing on patients with BPD with or without childhood traumatic experience and understanding the changes that occur in response to trauma. A detailed study of the impact of the nature and severity of trauma on children of various age groups may lead to a better understanding of how to modulate treatment based on individual needs. We need to understand and explore the risk of offspring developing BPD with epigenetic changes in parents. Future studies addressing whether intervention at an early age can halt or reverse any unwanted changes in the victim will play an essential role among patients of both sexes who may be at risk for BPD.
